# Supplementary material for: Identification of photoperiod-induced specific miRNAs in the adrenal glands of Sunite sheep (Ovis aries)
Source: Front Vet Sci. 2022 Jul 22;9:888207. doi: 10.3389/fvets.2022.888207 (PMC9354845; doi:10.3389/fvets.2022.888207)
Supplement: Supplementary file 1 [file Data_Sheet_1.ZIP › Supplementary file/miRDeep2 file.zip!/miRDeep2 file/Novel_Structure/SPLP42A2/Novel_281.pdf]

Provisional ID	:	NC_019458.2_2223
Score total	:	48.4
Score for star read(s)	:	3.9
Score for read counts	:	40.4
Score for mfe	:	2.5
Score for randfold	:	1.6
Score for cons. seed	:	
Total read count	:	91
Mature read count	:	88
Loop read count	:	0
Star read count	:	3

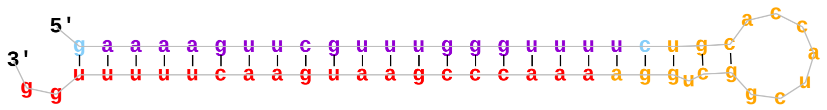

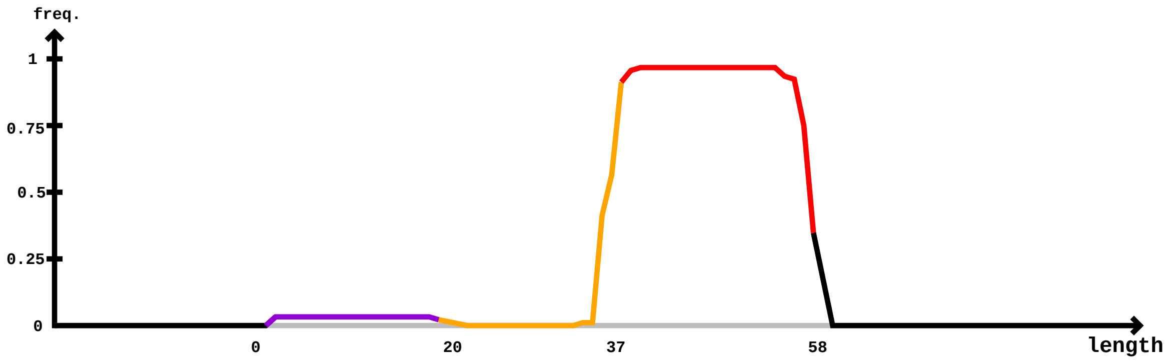

Star **Mature**

```
5'- cuggaacuauuggguuggccgaaaaguucguuuggguuuucugcaccaucggcuggaaaacccgaaugaacuuuuuggcgagcccgauaauucucgcaggaggaguuucuc  -3'  obs
    cuggaacuauuggguuggccgaaaaguucguuuggguuuucugcaccaucggcuggaaaacccgaaugaacuuuuuggcgagcccgauaauucucgcaggaggaguuucuc       exp
    ..((((((((((((((.(((((((((((((((((((((((((((.......)).)))))))))))))))))))))))))).))))))))).(((((....))))))))))..  reads  mm  sample
    .....................aaaaguucguuuggguu.........................................................................  1      0   seq
    .....................aaaaguucguuuggguuu........................................................................  1      0   seq
    .....................aaaaguucguuuggguuuG.......................................................................  1      1   seq
    .....................................................uggaaaacccAaaugaacuuu.....................................  1      1   seq
    .......................................................gaaaacccAaaugaacuuuuu...................................  5      1   seq
    .......................................................gaaaacccgaaugaacuuuuug..................................  10     0   seq
    .......................................................gaaaacccAaaugaacuuuuug..................................  21     1   seq
    .......................................................gaaaacccAaaugaacuuuuugg.................................  1      1   seq
    ........................................................aaaaccAgaaugaacuuu.....................................  1      1   seq
    ........................................................aaaaccAgaaugaacuuuu....................................  1      1   seq
    ........................................................aaaaccAgaaugaacuuuuu...................................  7      1   seq
    ........................................................aaaacccgaaugaacuuuuG...................................  2      1   seq
    ........................................................aaaacccUaaugaacuuuuu...................................  1      1   seq
    ........................................................aaaaccGgaaugaacuuuuugg.................................  1      1   seq
    ........................................................aaaacccgaaugaacuuuuugg.................................  1      0   seq
    .........................................................aaacccgaaugaacuuu.....................................  1      0   seq
    .........................................................aaacccgaaugaaAuuuuug..................................  1      1   seq
    .........................................................aaaccAgaaugaacuuuuug..................................  1      1   seq
    .........................................................aaacccgaaugaacuuuuuC..................................  1      1   seq
    .........................................................aaacccgaaugaacuuuuugg.................................  28     0   seq
    ..........................................................aacccgaaugaacuuuuu...................................  1      0   seq
    ..........................................................aacccgaaugaacuuuuug..................................  2      0   seq
    ..........................................................aacccgaaugaacuuuuuC..................................  1      1   seq
    ...........................................................acccgaaugaacuuuuuggc................................  1      0   seq
```
